# Model-Based Experimental Development of Passive Compliant Robot Legs from Fiberglass Composites

**DOI:** 10.1155/2015/754832

**Published:** 2015-06-01

**Authors:** Shang-Chang Lin, Chia-Jui Hu, Wen-Pin Shih, Pei-Chun Lin

**Affiliations:** Department of Mechanical Engineering, National Taiwan University, Sec. 4, No. 1 Roosevelt Road, Taipei 106, Taiwan

## Abstract

We report on the methodology of developing compliant, half-circular, and composite robot legs with designable stiffness. First, force-displacement experiments on flat cantilever composites made by one or multifiberglass cloths are executed. By mapping the cantilever mechanics to the virtual spring model, the equivalent elastic moduli of the composites can be derived. Next, by using the model that links the curved beam mechanics back to the virtual spring, the resultant stiffness of the composite in a half-circular shape can be estimated without going through intensive experimental tryouts. The overall methodology has been experimentally validated, and the fabricated composites were used on a hexapod robot to perform walking and leaping behaviors.

## 1. Introduction

After millions of years of adaptation in the natural environment, animal legs have evolved and diversified their leg morphology into various forms. Though the appearance may be different, the main function of the legs still lies in enabling animals to negotiate widely diversified natural terrains. Thus, how the individual leg moves and how the legs are coordinated are both critical and fundamental biomechanics questions. Previously, the researchers found that, no matter how many legs the biological systems have, their dynamic running locomotion in the sagittal plane can be approximated by a simple mathematical model, “SLIP” (Spring-Loaded Inverted Pendulum) [1–3]. The SLIP model is composed of a point mass representing the body and a massless spring leg. The SLIP model is widely recognized as the intrinsic and qualitative “template” which can describe the general running locomotion of legged animals with different geometrical shapes and evolutionary stages as the “anchor” [4].

The research described in the previous paragraph suggests that the legs of bioinspired legged robots should be operated like a passive and linear spring by using stiffness or force control. For example, on the arms [5], hands [6], legs [7], or exoskeleton [8]. Though ideally the spring-like behavior can be achieved by controlling multi-degree-of-freedom (DOF) legs to act like a spring, empirically this approach is extremely challenging because artificial actuators such as electric motors have limited power density in comparison to biological actuators such as muscles. Thus instead of using the animal-like multi-DOF legs, some legged robots use passive compliant components as legs [9]. The quadruped Scout series uses linear springs as the legs [10–12]. The hexapod RHex series went through various generations of legs [13, 14], and it uses the half-circular legs made of fiberglass composite in its latest version [15, 16]. The hexapod Sprawl series uses polyurethane to generate passive compliance of the legs [17, 18].

The material in a half-circular shape is one of the ideal components for compliant legs on a robot owing to its simple morphology. The RHex with the half-semicircular legs has demonstrated versatile behaviors such as running [19], stair ascent/descent [20, 21], high-step climbing [22], bounding [23], leaping [24], and other advanced dynamic maneuvers [16]. The RHex uses a fiberglass composite as the leg material because commercial, deformable polymers are unlikely to meet the necessary requirements such as stiffness, robustness, and minimum plastic effect. However, making a fiberglass composite leg with adequate stiffness and in a circular shape still relies heavily on the engineering trial-and-error process.

Here, we propose a methodology to design and fabricate a compliant half-circular fiberglass composite with desired stiffness. The composite made in strips is utilized as the reference test to investigate the empirical elastic properties because a flat composite sheet is easier to fabricate and each sheet can produce strips with different fiber orientations for evaluation. The equivalent elastic modulus is adopted as the interface to link the microscale mechanics to the macroscale resilient behaviors because the detailed mechanics of the fiberglass composite as determined by the mechanics of the laminates and bonding adhesives and their interactions are hard to analyze analytically. In order to link the elastic behaviors of the flat sheets and the half-circular beams, two models that can be experimentally implemented are introduced to link the resultant stiffness to the equivalent elastic modulus. As a result, the mechanical properties derived based on the deformation test on the flat strip composite can be directly deployed to the half-circular composite.

The remainder of the paper is organized as follows: Section 2.1 introduces the elastic models utilized for experimental implementation, while Section 2.2 describes the experimental methods.  Section 3 report the experimental results of the cantilever and half-circular composites.  Section 4 concludes the work.

## 2. Materials and Methods

### 2.1. Elastic Models

In general, the resultant stiffness of a material is determined by its elastic modulus as well as its geometric dimensions. From the aspect of robotic engineering, where the system is usually integrated with existing materials, the changing of material properties to fit the robotic requirements is usually a challenge. Thus the required mechanical characteristics of the robot are usually achieved by designing and tuning the dimensions of the components and choosing adequate and available raw materials. For example, in our application where compliant legs are desired, the design strategy lies in investigating the fiber weave pattern and the number of layers of fiberglass sheets required to form the leg, so the macroscale leg stiffness can be achieved.

A compliant leg in a half-circular shape is desired for two reasons: first, a robot with half-circular legs has rolling contact to the ground, which is reported to have excellent locomotion characteristics. Second, the component in half-circular shape is easy to fabricate and has compliant behavior. Since the majority of the reported analyses are on flat sheets/bars (i.e., beam model), this work starts with the analysis on the flat materials first and then extends to the half-circular shaped materials.

#### 2.1.1. Cantilever Beam Model

**Figure 1:**
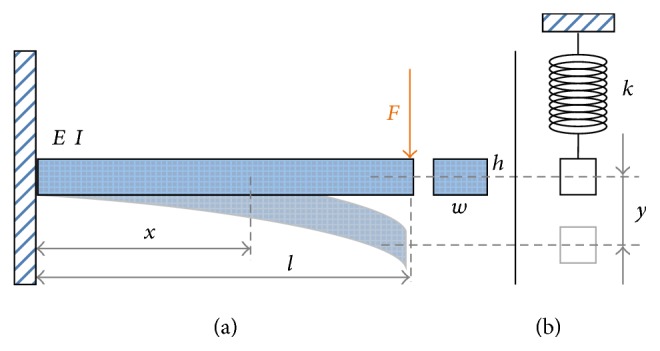
The solid mechanic model (a) and the simplified model (b) of the cantilever beam.

The elastic characteristics of the cantilever rectangular beam model are shown in Figure 1(a) and are described as follows. It has one elastic parameter, the elastic modulus (*E*), and three geometric parameters, including length (*l*), width (*w*), and thickness (*h*). When the force (*F*) is applied to the beam, the free end of the beam will deform a distance (*y*). According to the solid mechanics of materials, the deformation of the beam mainly results from the tension and compression of the beam material in microscale. The normal strain in any rectangular cross section of the beam can be represented as

(1) $$\begin{array}{c} \sigma =\frac{M\cdot c}{I}, \end{array}$$

where *c*, *M*, and *I* represent the distance from the neutral surface, the bending moment, and the moment of inertia, respectively. The last term is determined by the width (*w*) and height (*h*) of the beam, *I* = *wh* ^3^/12. The bending moment (*M*) caused by the external forced loading on an arbitrary position of the beam can be expressed as

(2) $$\begin{array}{c} M=F\left(\;l-x\;\right). \end{array}$$

The strain energy of the whole beam (*U*_*b*_) due to the moment can then be computed

(3) Ub∬σ dϵ dV=∫0LM22EIdx=∫0LFl−x22EIdx=F2l36EI.

The strain energy from shear force is ignored because it is relatively small in comparison to that from bending. In addition, the elastic property of the cantilever beam model can be approximated by a lumped linear spring with stiffness (*k*), as shown in Figure 1(b), whose equilibrium position resides at the position where the beam is not force loaded. The spring potential of the lumped model is

(4) $$\begin{array}{c} U_{k}=\frac{1}{2}ky^{2}. \end{array}$$

Because the cantilever beam model and the lumped model represent the same system, the potential energies of both systems can be treated the same

(5) $$\begin{array}{c} U_{b}=\frac{F^{2}l^{3}}{6EI}=\frac{{\left(ky\right)}^{2}l^{3}}{6EI}=\frac{1}{2}ky^{2}=U_{k}. \end{array}$$

As a result, the “resultant stiffness” of the beam model can be derived as

(6) $$\begin{array}{c} k=\frac{3EI}{l^{3}}=\frac{wh^{3}E}{4l^{3}}. \end{array}$$

#### 2.1.2. Curved Beam Model

**Figure 2:**
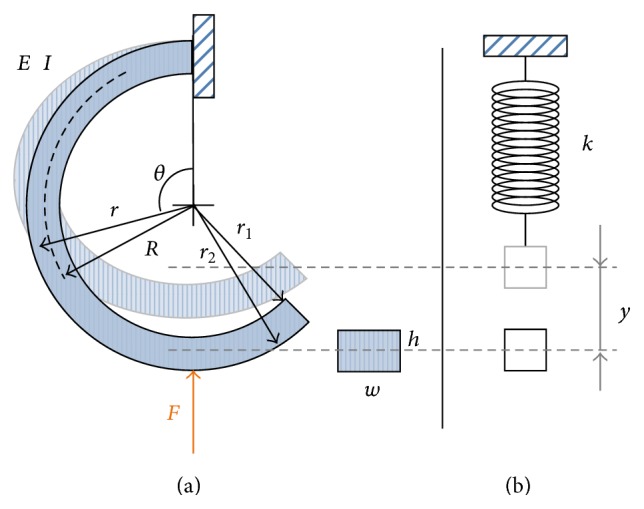
The solid mechanic model (a) and the simplified model (b) of the curved beam.

The cantilever rectangular beam model can be extended to the model with a half-circular shape as shown in Figure 2(a), the same shape as the robot leg. The curved beam also has four parameters: the elastic modulus (*E*), the radius of the curvature (*R*), the beam width (*w*), and the beam thickness (*h*). While an external force is applied to the beam at the bottom side, the arbitrary cross section at angle *θ* has a normal force (*F*_*θ*_) and a bending moment (*M*_*θ*_):

(7) Fθ=Fsin⁡θ,Mθ=FRsin⁡θ.

Thus, the normal stress at this position can be computed:

(8) σθ−FθA+Mθr−RI=−Fsin⁡θA+FRsin⁡θr−RI,

where *A* represents the cross section area and *r* is the distance from the circular center to the stress. The strain energy of the whole half-circular leg (i.e., from *θ* = 0° to *θ* = 180°) can be yielded:

(9) Uc=∭σθ2EdV=w2E·∫0π∫r2r1−Fsin⁡θA+FRsin⁡θr−RI2rdr dθ=wF2π4Er12−r222A2−2RAI13r13−r23−R2r12−r22+R2I2r14−r244−2Rr13−r233+R2r12−r222,

where the symbols *r*_1_ and *r*_2_ represent the outer and inner radii of the curved beam, respectively. As shown in Figure 2(a), these two radii can be related to the dimensions of the beam by

(10) r1−r2=h,r1+r2=2R.

By importing (10) into (9), the strain energy of the curved beam can be derived as

(11) Uc=wF2π4ERhA2−2AIRh33R2+h24−R3h+R2I2Rh22R2+h22−2Rh33R2+h24+R3h=F2Rπ4whE12R2h2−1.

Similarly, the elastic property of the curved beam model can be approximated by a lumped linear spring with stiffness (*k*), as shown in Figure 2(b), whose equilibrium position resides at the point where the curved beam is not force loaded. The spring is vertically posed with a natural length of 2*R*. Since the curved beam model and the lumped model represent the same system, the strain energies of both systems can be treated the same:

(12) UcF2Rπ4whE12R2h2−1=ky2Rπ4whE12R2h2−1=12ky2=Uk.

As a result, the “resultant stiffness” of the curved beam model can be derived as

(13) $$\begin{array}{c} k=\frac{2whE}{R\pi \left(12R^{2}/h^{2}-1\right)}. \end{array}$$

Because the ratio *R*/*h* of the curved beam in our robot leg application is greater than an order, the constant term 1 in the parenthesis can be ignored. Therefore, the resultant stiffness can be approximated as

(14) $$\begin{array}{c} k=\frac{wh^{3}E}{6R^{3}\pi }. \end{array}$$

The approximation also indicates that the strain energy caused by normal force is much smaller in comparison to that caused by bending moment, similar to the results of the cantilever beam model as reported.

#### 2.1.3. Composite Model

**Figure 3:**
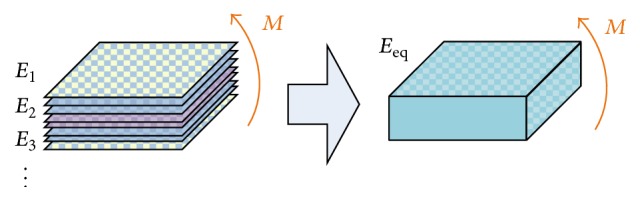
The composite material is stacked in layers of materials with different elastic moduli, and the composite can be regarded as a homogeneous material with an “equivalent” elastic modulus.

Equations (6) and (14) reveal the relation of the model's resultant stiffness to its geometric parameters and elastic modulus. This modulus represents the explicit behavior of the complex internal stress-strain behavior. The beam formed by composite materials is one of the representative examples. As shown in Figure 3, assume the beam is formed by layered thin sheets and that the stacking is symmetric to the neutral surface of the beam. Owing to the geometrical constraint, the strain (*ε*_*i*_) within each layer and between the layers is continuous, but the stress at the interface of the layers can be varied. Similar to the behaviors observed in the cantilever beam model and curved bean model, the bending moment is the main factor that determines the resultant stiffness. This is mainly caused by the moment in the cross section. Thus, the bending moment of the composite beam model can be computed as the sum of all moments caused by the normal stress in all layers:

(15) M=∬σy dA=2w∫0d1σ1y dy+⋯+∫dn−2dn−1σn−1y dy+∫dn−1dnσny dy=2w∫0d1E1Eny2hσndy+⋯+∫dn−2dn−1En−1Eny2hσndy+∫dn−1dnEnEny2hσndy=2σnwEnhE1∫0d1y2dy+⋯+En−1∫dn−2dn−1y2dy+En∫dn−1dny2dy=2σnw3Enh·∑i=1nEidi3−di−13=2εnw3h∑i=1nEidi3−di−13.

Then, the “equivalent” elastic modulus of this composite beam can be computed:

(16) M2εnW3h∑i=1nEidi3−di−13=2EeqεnW3h·h3Eeq∑i=1nEidi3−di−13h3.

The derivation shown above suggests a methodology to develop a compliant robot leg from composite materials. In applications of uniform and isotropic materials where the material dimensions and elastic modulus are known, (6) and (14) reveal the effects of these parameters on the overall compliant behavior, the “resultant” stiffness. In contrast, in our application of composite materials where the “equivalent” elastic modulus is not a given parameter, (6) and (14) can be reversely utilized to compute the equivalent elastic modulus, where the detailed mechanics of the stress-strain behavior are not necessary to the analysis. In this case, both the dimensions (i.e., (*l*, *w*, *h*) or (*R*, *w*, *h*)) and resultant stiffness (*k*) of the beam should be known a priori. The former is usually known when the material is fabricated. The latter can be obtained by empirical force-displacement measurement, and the detailed method for this will be reported in the next section.

### 2.2. Experimental Methods

#### 2.2.1. Fiberglass Sample Preparation

The fiberglass composite is formed by layered fiberglass cloths with epoxy in-between. The elastic modulus of each thin fiberglass layer is determined not only by the mechanical properties of the glass fiber itself but also by how the fibers are woven. The anisotropic properties of the fibers are actually favorable because different mechanical properties of the composite can be achieved by the sample without altering its size. To investigate the isotropic effect, two kinds of fiberglass cloths were adopted, the E-glass (0°) and the E-glass (−45°, 0°, 45°), and Table 1 shows their specifications.

**Figure 4:**
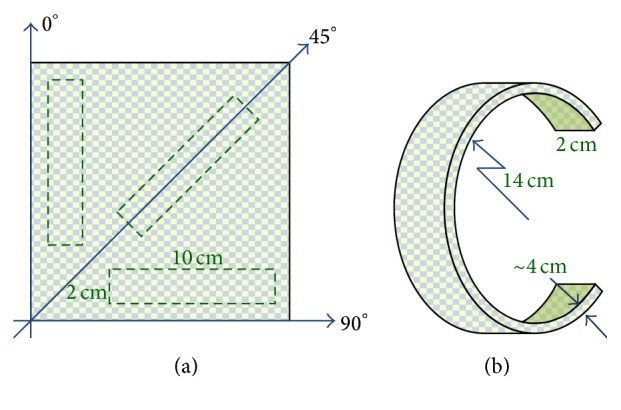
The fiberglass composites: (a) the definition of the cutting directions and the size of the strip samples. (b) The size of the curved beam sample.

The fiberglass composites were fabricated in two different shapes for experimental evaluation. The first shape is a strip some 10 cm long and 2 cm wide. In addition, the fiberglass cloths were cut in three different directions (0°, 45°, 90°) as shown in Figure 4(a), to evaluate the anisotropic effect. The second shape is the half-circular shape shown in Figure 4(b), the same shape as the legs on the robot.

**Figure 5:**
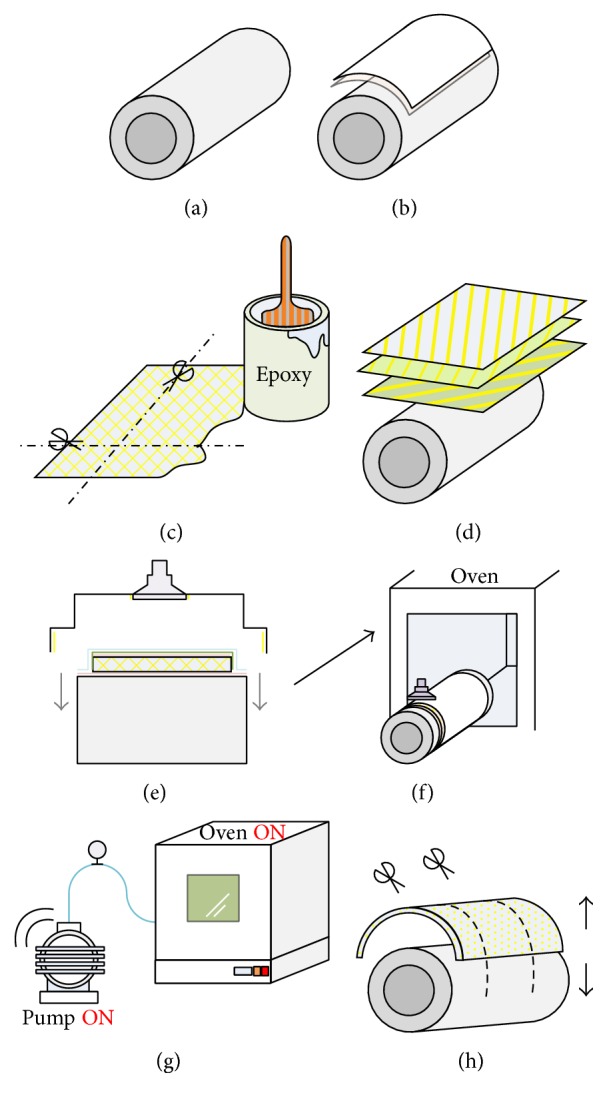
The fabrication process for the fiberglass composites.

The step-by-step fabrication procedure for the half-circular leg is described in the exemplary demonstration. (i) Preparing a mold as shown in Figure 5(a): the fiberglass cloths were stacked from the inner to outer surfaces, so a mold supporting the shape of the inner surface is required. After determining the dimensions of the leg, an aluminum hollow cylinder was made as the mold. (ii) Covering a release film and a peel-ply on the mold is as shown in Figure 5(b). (iii) Preparing fiberglass cloths as shown in Figure 5(c): cut the cloths to the right dimensions following the selected directions. (iv) Preparing the epoxy resin is as shown in Figure 5(c). (v) Stacking the fiberglass cloths is as shown in Figure 5(d): brushing the cloths with epoxy and stacking them layer by layer on top of the peel-ply. After each layer was stacked, using a roller to roll the surface in order to tightly compress the layered composite and to force resin into the cloth. The type and orientation of the anisotropic cloth in each layer can be different, determined by the experimental setting. (vi) Covering a release film and a perforated film on the stacked fiberglass layers. (vii) Covering a breather ply, so the vacuum pump can remove the air bubbles within the stacked fiberglass layers. (viii) Covering a vacuum bagging film and sealing it with a sealant tape are as shown in Figure 5(e). (xi) Curing the composite in the oven with the vacuum pump on is as shown in Figures 5(f) and 5(g). The temperature and time for curing are determined by the epoxy properties. (x) After curing, the raw composite was fabricated and ready for cutting into the shape of the legs as shown in Figure 5(h).

**Figure 6:**
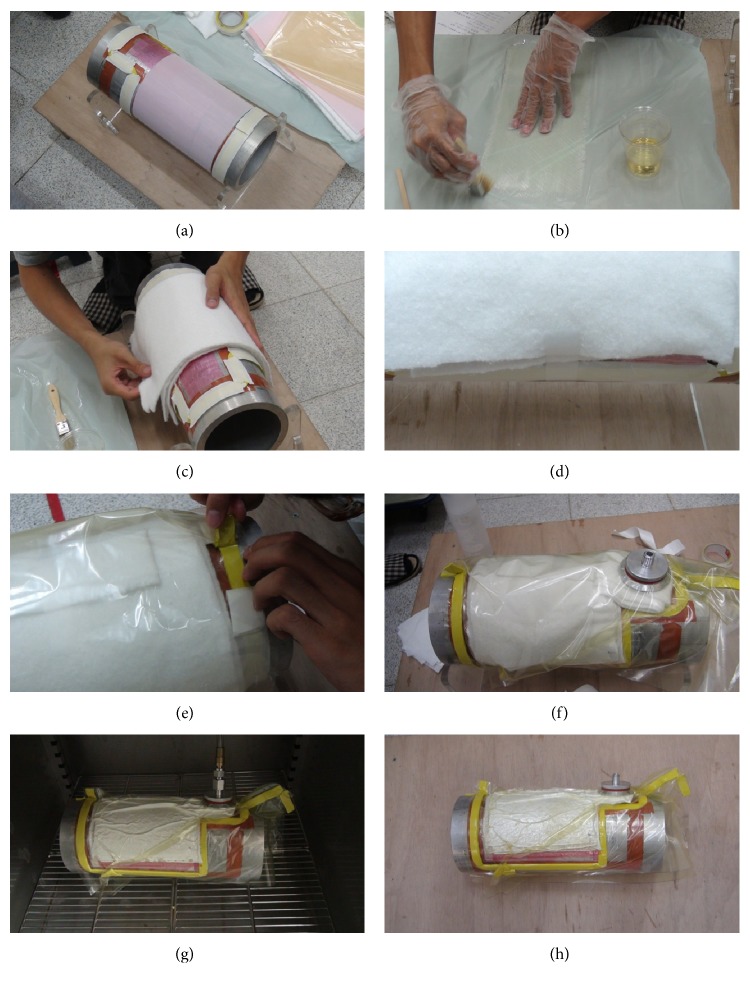
Photos of the fabrication process for the fiberglass composites.

Figure 6 shows various photos taken during the fabrication process. (a) The release film and the peel-ply were covered on the mold. (b) The cloths were brushed with the epoxy. (c) The release film, perforated film, and breather ply were covered on the stacked fiberglass layer. (d) The edges of all layers were trimmed. (e) The vacuum bagged film was covered and sealed the material assembly by using the sealant tape. (f) A photo of the whole assembly which contains the fiberglass layers and other associated films. (g) The assembly was cured in the oven. (h) A photo of the whole assembly after curing.

#### 2.2.2. Sample Stiffness Measurement

Customized testing setups were built to take the force-displacement measurements of the cantilever beam and curved beam, where the force and displacement were directly matched with the operational directions of the virtual springs as shown in Figures 1(b) and 2(b). Though the conventional tensile test can also derive the elastic modulus of the material, the setup is hard to modify for our force-displacement measurements. In addition, the layered composite material has different behaviors in tension and compression, so the customization is required for experimental validation.

**Figure 7:**
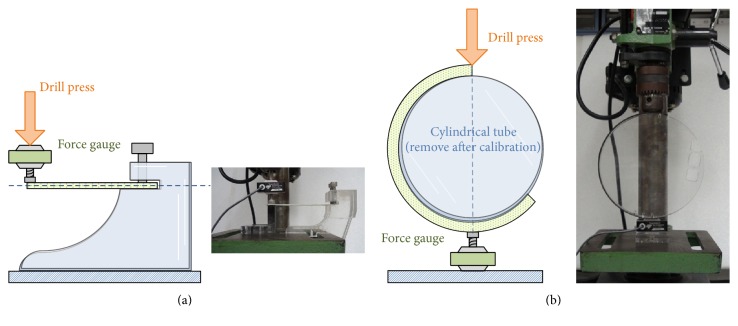
Measurement setup of the cantilever beam experiments (a) and the curved beam experiments (b).

The sample was clamped to the testing setup as shown in Figure 7. The setup was mounted under a drill press, and the one-dimensional compression motion was generated by the linear guide way of the drill press. By using the locking mechanism of the drill press, the sample was set to be compressed with several displacements. In the meantime, the force was measured using a commercial bidirectional force transducer.

The experiment procedure has three steps. (i) Calibration: for the rectangular beam, use the jig structure to hold the cantilever beam horizontally as shown in Figure 7(a). Then vertically align the drill press and force sensor to the free end of the sample, so the force can be applied to the sample with displacement in the correct direction. For the curved beam, mount the cylindrical tube inside the curved beam, as shown in Figure 7(b), and place the assembly in between the force sensor at the bottom and the drill press at the top. Make sure the force can be applied to the sample with displacement in the correct direction. After the alignment, remove the cylindrical tube circular, so the curved beam can be subject to compression. (ii) Measurement: the force data is collected when the displacements of the rectangular beam are 5 mm, 7 mm, and 10 mm, and when those of the curved beam are 3 mm, 5 mm, 7 mm, 10 mm, and 12 mm. The experiments are repeated several times using different samples with the same parameters, so the variation of the mechanical characteristics of the samples has less effect on the final results. (iii) Analysis: use the linear regression method to find the slopes of the force versus displacement data, which represents the “resultant” stiffness of the rectangular beam or the curved beam. Then, by using (6) and (14), the “equivalent” elastic modulus of the beam can be derived.

## 3. Results and Discussion

### 3.1. Experiment Results of the Strip Fiberglass Composites in the Cantilever Beam Test

The equivalent elastic modulus of the fiberglass composite is determined by several factors such as the elastic modulus and weave of the fiberglass cloths, the elastic modulus of the epoxy, and the stacking methods. To simplify the development, the microscale mechanics are ignored but the macroscale mechanics of the composite are captured. More specifically, the equivalent elastic modulus serves as the key factor for evaluating the performance of the layered composite.

#### 3.1.1. Experiments on 4-Layer Composites Made by One Kind of Fiberglass Cloth

**Figure 8:**
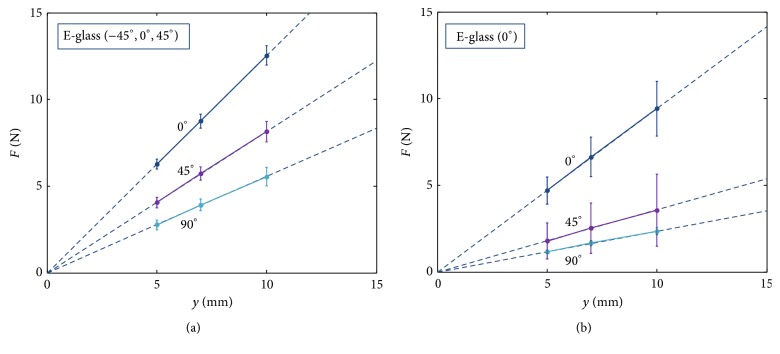
The force-displacement relation of the fiberglass composite strips made by the E-glass (−45°, 0°, 45°) in (a) and the E-glass (0°) in (b).

**Figure 9:**
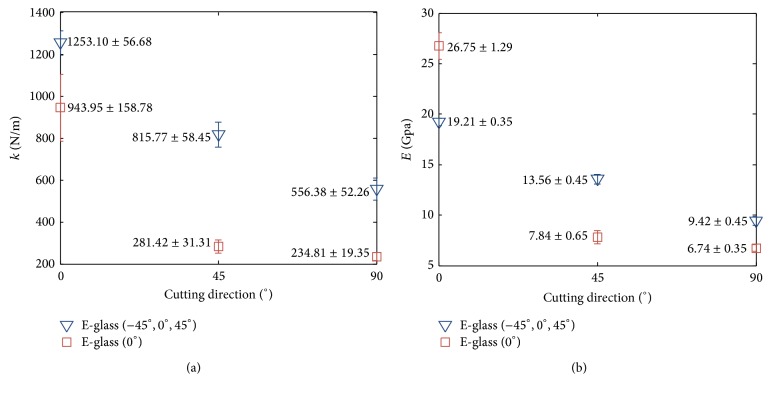
The resultant stiffness (a) and equivalent elastic moduli (b) of the fiberglass composite strips made by the E-glass (−45°, 0°, 45°) and the E-glass (0°) with three different cutting directions.

The fiberglass composite strips were fabricated by a single kind of fiberglass cloth and in four layers. Together with three different cutting directions (0°, 45°, 90°), there are six kinds of samples in total.  Table 2 lists forces of the strips in three displacements.  Figure 8 shows the force-displacement plot of these samples. The data is represented in a statistical manner with means and standard deviations (STD), which are obtained from seven experimental runs. Several comments can be addressed:

(i) The means of each kind of composite are aligned to nearly a straight line which passes zero, indicating that it is reasonable to approximate the force-displacement of the cantilever beam by a virtual spring system as shown in Figure 1.
(ii) The stiffness of the virtual springs (i.e., slope of the force-displacement plot) shown in Figure 8 can be extracted and replotted as the vertical axis as shown in Figure 9(a). The figure clearly shows that the stiffness of the E-glass (−45°, 0°, 45°) is larger than that of the E-glass (0°), no matter what the cutting direction is. It may be intuitive that the stiffness of the E-glass (0°) with the cutting direction in 0° (i.e., hereafter referred to as E-glass (0°)-0°) should be larger than that of the E-glass (−45°, 0°, 45°) in any cutting direction because the fibers of the former are more aligned to resist the bending. However, because the E-glass (−45°, 0°, 45°) has 20% more knitted yarn per square meter than the E-glass (0°), the composite of the former is thicker than that of the latter, though both have the same four layers. According to (6), the resultant stiffness is affected not only by the elastic modulus but also by the geometric properties of the composite. In this set of experiments, the thickness of the composite appears to have a larger effect than the fiber direction, resulting in the phenomenon shown in Figure 8.
(iii) The equivalent elastic moduli of the composites can be computed by (6) with given dimensions. As shown in Figure 9(b), after eliminating the geometric effects, the elastic modulus of the E-glass (0°)-0° has the highest value, as expected. Furthermore, the elastic moduli of the E-glass (0°)-45° and E-glass (0°)-90° have very low values, and this phenomenon is also expected because few fibers are aligned in these directions. In contrast, the elastic modulus of the E-glass (−45°, 0°, 45°) has a gentle change.

In short, this set of experiments confirms that (i) the mechanic behavior of the empirical composite samples matches that of the model and (ii) the idea of using the virtual spring to model the elastic behavior is feasible in this composite case. In addition, the elastic moduli of these two kinds of cloths, each with three cutting directions, are yielded and will serve as the reference for the following development where the composite is composed of both fiberglass clothes.

#### 3.1.2. Experiments on 4-Layer Composites Made by Two Kinds of Fiberglass Cloths

In this set of experiments, two kinds of fiberglass cloths are mixed to form a 4-layer composite. Four different stacking combinations of the composite were used, and all were symmetric to the center plane. The (S) stacking configuration used the E-glass (0°) and the E-glass (−45°, 0°, 45°) with the same orientation as the inner layer and outer layer, respectively. The (D) stacking configuration used the E-glass (0°) as the inner layer and the E-glass (−45°, 0°, 45°) as the outer layer, but the latter rotated 90°. The (S′) and (D′) stacking configurations had reversed inner and outer fiberglass clothes without altering the cutting directions accordingly. These four configurations were adopted in an attempt to evaluate two effects: first, the positions of the layers in the whole composites (i.e., S versus S′ and D versus D′) and, second, the effect of the cutting direction on the stiffness.

**Figure 10:**
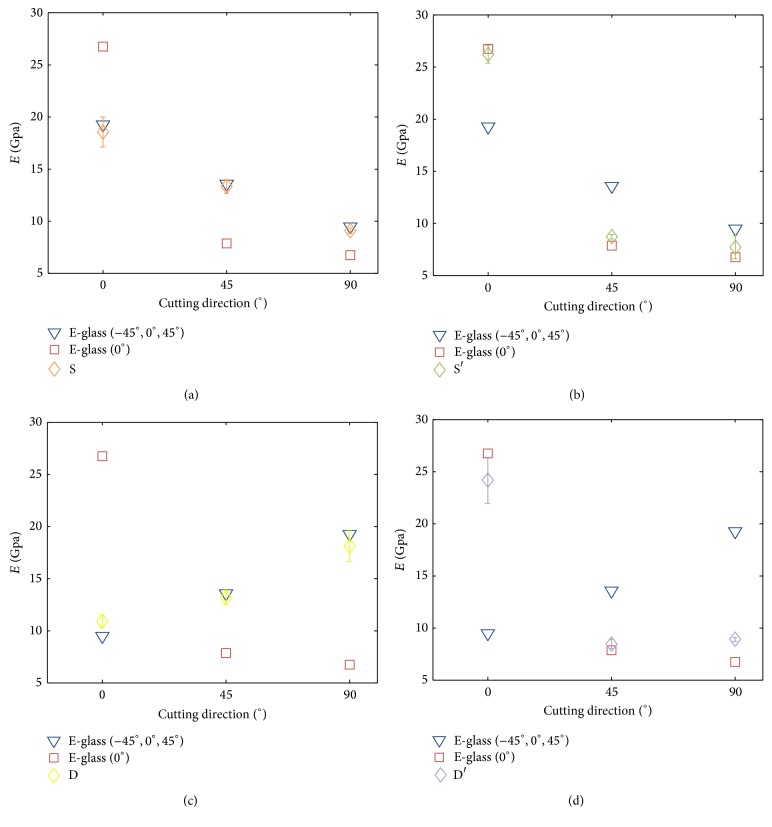
The equivalent elastic moduli of the 4-layer fiberglass composite strips made by the mixed E-glass (−45°, 0°, 45°) and the E-glass (0°) in four different layer configurations (S, S′, D, D′).

Figure 10 plots the equivalent elastic moduli of these four configurations in statistical representations (i.e., mean and STD). In addition, the equivalent elastic modulus of the E-glass (0°) and the E-glass (−45°, 0°, 45°) with the same cutting directions are also plotted for comparison. The figure reveals that, as expected, the elastic moduli of the mixed four-layer composites are mostly located between those of the four-layer composite made either by the E-glass (0°) or the E-glass (−45°, 0°, 45°) with the same cutting directions. Moreover, the elastic moduli of the four-layer composites are closer to that of the outer layer than the inner layer because the bending-induced deformation is mainly determined by the moment generated by the outer layer.

The equivalent elastic modulus of the mixed composite can also be estimated by (16), where the dimensions of the composite were empirically measured and the elastic moduli of the individual layers were derived by the experiment described in Section 3.1.1.  Table 3 shows the elastic moduli of four configurations by prediction and three test experiments. The table reveals that, except for one test result, the percentage errors between the estimated and experiment results of 36 tests were within 10%, and 23 out of 36 tests had errors less than 5%. The averaged error of eleven out of twelve types of composites is less than 4%. The matched result confirms that the composites made by different fiberglass clothes with different cutting directions can be empirically made, and its equivalent elastic modulus can also be predicted with reasonable accuracy.

#### 3.1.3. Experiments on 6-Layer Composites Made by Two Kinds of Fiberglass Cloths

**Figure 11:**
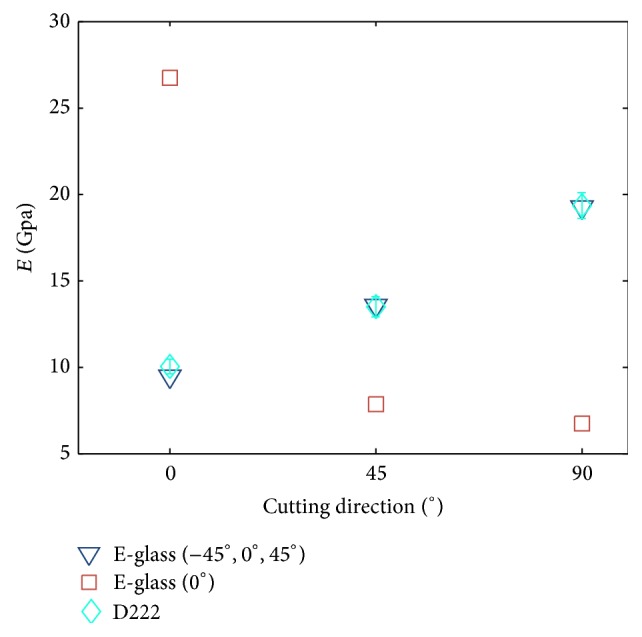
The equivalent elastic moduli of the 6-layer fiberglass composite strips made by the mixed E-glass (−45°, 0°, 45°) and the E-glass (0°) in D222 layer configurations.

The estimation of the elastic modulus of the composite stacked by the E-glass (0°) and the E-glass (−45°, 0°, 45°) is functional not only for the 4-layer composite but also for other numbers of layers. A 6-layer (D222) stacking configuration used the E-glass (0°)-0° as the inner two layers and the E-glass (−45°, 0°, 45°)-90° as the outer four layers was made to confirm this conclusion.  Figure 11 shows the equivalent elastic modulus of this mixed composite as well as that of the composites made by each kind of fiberglass cloth. As expected, because the outer layer dominates the elastic behavior, the equivalent elastic modulus of the mixed composite is close to that of the E-glass (−45°, 0°, 45°). In addition, because four out of six layers were E-glass (−45°, 0°, 45°), the equivalent elastic modulus is closer to that of the E-glass (−45°, 0°, 45°) than the composite of the 4-layer D stacking configuration.  Table 4 shows the elastic modulus of the mixed composite by prediction and three test experiments. The table reveals that the percentage errors between the estimated and experiment results of 9 tests were within 10%, and 6 out of 9 tests had errors less than 5%.

The matched predicted and experimental results suggest that the methodology can be adopted as a useful design tool: if the equivalent elastic modulus of the individual layer is known a priori, the desired elastic modulus of the composite can be correctly designed in simulation first without relying on an experimental trial-and-error method. This methodology will be extended to design compliant half-circular legs in the next section.

### 3.2. Experiment Results of the Curved Beam Model

The ultimate goal of this work is to develop a methodology for fabricating a fiberglass composite that has the desired resultant stiffness, so the composite can be implemented on a robot for developing dynamic behaviors. In order to link the development of the half-circular composite to the strip composite described in the previous section, the half-circular composite for experimental validation used the same two kinds of the fiberglass cloths as well as the same layer configuration. Unlike the composite strips where the samples in all three cutting directions can be obtained by cutting a single large composite plate, the layers of the half-circular composite can only have one specific configuration at a time. As a result, instead of making composites in all twelve combinations of (S, S′, D, D′), only the four of them closest to our interest were selected for evaluation, including S-0°, S-90°, D-0°, and D-90°. In addition, the composites made by one kind of fiberglass cloth were made for comparison as well, including E-glass (0°)-0°, E-glass (−45°, 0°, 45°)-0°, and E-glass (−45°, 0°, 45°)-90°.

**Figure 12:**
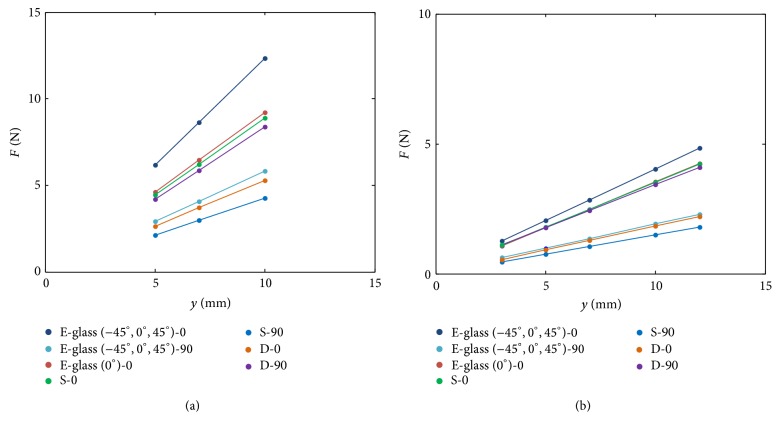
The force-displacement relation of the fiberglass composite strips (a) and semicircular composites (b) made by the E-glass (−45°, 0°, 45°) and the E-glass (0°) in seven different material and layer configurations.

Figure 12 shows the force-displacement of the composite strips and the half-circular composites. Similar to the behavior observed in the composite strip, the means of the measured experimental data of the half-circular composite are aligned nearly to a straight line. Thus it is reasonable to approximate the force-displacement of the half-circular composite by a virtual spring system as shown in Figure 2. By using the stiffness (i.e., slope of this figure) and measured dimensions, the equivalent elastic modulus of the half-circular composite can be computed using (14).  Table 5 lists the elastic moduli of these configurations by experimental measurement and estimation, where the latter was obtained by the results of composite strips. The close match between these two data sets in most of the composite configurations confirms that the fabricated strip and half-circular composites with the same layer configuration have similar values of elastic modulus, and this further confirms two points: first, the fabrication process and product quality are reliable, and, second, the test results and model development of the composite strips can provide useful design information before making half-circular composites.

The RHex-style robot in our lab has a mass of 6.5 kg and the dimensions 0.45 m in length, 0.28 m in width, and 0.2 m in height (standing height). The current robot legs have radius 70 mm and width 20 mm. In order to evaluate the effect of leg compliance on the robot's behavior, the new composite legs should have the same dimensions as the old legs. Thus, according to (14), the possible parameters for variation are the thickness and equivalent elastic modulus of the composite. Thus the resultant stiffness can be varied by changing either cloth type or the number of layers.

The desired stiffness of the robot can be estimated by the dynamics of the spring-mass model as reported in [25]. The literature reported that animals with different masses have different stride frequencies [26]. A system weighing around 6.5 kg should have a stride frequency within the range of 2–4 Hz. Note that because the robot utilizes an alternating tripod gait, the leg stride frequency should be half of the model stride frequency, about 1-2 Hz. In addition, because tripod locomotion involves three legs contacting the ground simultaneously, the stiffness of each leg should be one-third of the model stiffness. As a result, the individual leg stiffness is set to about 2000–2500 N/m.

In the first attempt, the fiberglass legs were made by the E-glass (0°, 90°)-0°. As shown in Table 6, the legs have an averaged resultant stiffness of 2591 Nm, 3.6% error to the desired 2500 N/m. However, when the legs were installed on the robot for the running test, the low lateral stiffness resulted in lateral motion disturbance, so the robot could hardly perform straight forward locomotion. Thus, in addition to planning the compliance in the sagittal plane of the robot, the lateral stiffness should be considered. This is the reason why we need to stack the different fibers together.

**Figure 13:**
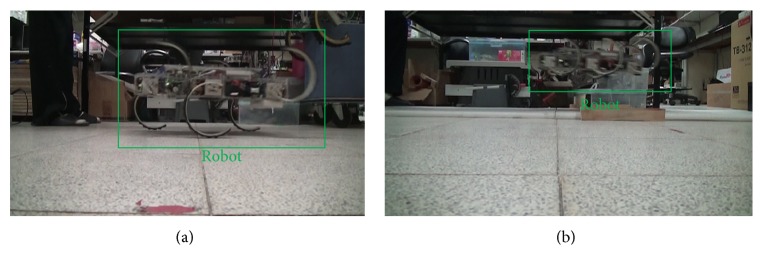
The robot installed with the fiberglass composite legs performs walking (a) and leaping (b) behaviors.

In the second attempt, the fiberglass legs were made by three layers of E-glass (−45°, 0°, 45°)-90° on each outer side and four layers of E-glass (0°)-0° on the inner side, effectively the “D configuration” but with more layers. The design process followed that described in the previous two sections. As shown in Table 7, the legs have the averaged resultant stiffness of 2555 Nm, 2% error to the desired 2500 Nm. In addition, because of the 90° rotated configuration of the E-glass (−45°, 0°, 45°), the lateral stiffness as well as the torsional stiffness with respect to the vertical axis are stiffer to resist the perturbation generation during locomotion test. The robot with this set of legs can perform walking and leaping locomotion, with the snapshots extracted from the recorded video being shown in Figure 13 as a demonstration.

## 4. Conclusion

We report on the methodology for developing compliant, half-circular, and composite robot legs with designable stiffness. The composite made in strips is utilized as the reference test to investigate the empirical elastic properties because the flat composite sheet is easier to fabricate and each sheet can produce strips with different fiber orientations for evaluation. By executing the force-displacement experiments on the flat cantilever composite composed of the same kind of fiberglass cloth, together with the mapping model from the cantilever mechanics to the reduced-order virtual spring, the equivalent elastic modulus of the composite can be revealed. In this work, two kinds of fiberglass cloths, each with three cutting directions, were tested, so there are six reference elastic moduli to serve as the “database” for designing composites with mixed fiberglass cloths. The 4-layer composites with twelve configurations were experimentally evaluated, where the equivalent elastic moduli of the estimated and experimental values were utilized as the comparison basis. The results reveal that, among 36 tests, 35 of them have percentage errors less than 10%, and 23 of them have percentage errors less than 5%. The 6-layer composites with three configurations were evaluated as well. The results reveal that, among 9 tests, all have percentage errors less than 10%, and 6 out of 9 tests have percentage errors less than 5%. Qualitative observation reveals that the fiber directions of the layered cloth have a critical effect on the equivalent elastic modulus. As expected, the cloths at the outer layers had the larger effect on the equivalent elastic modulus.

After confirming that the elastic behavior of the flat composites made by mixed fiberglass layers can be correctly estimated, the strategy is extended to the composite in half-a-semicircular shape. The mapping model from the curved beam mechanics to the reduced-order virtual spring was developed to map the equivalent elastic modulus back to the resultant stiffness of the half-circular composite. The experimental results confirm that the designed 8-layer half-circular composites have an averaged resultant stiffness of 2555 Nm, which has only a 2% error to the desired stiffness value 2500 Nm. The fabricated composites were utilized as the robot legs, and the robot can reliably perform walking and leaping behaviors.

We are currently in the process of revising the methodology to include the effect of damping into the design process. This would require model formation in a dynamic manner, and the experimental setup should be capable of capturing the dynamics of the composites as well.

## Figures and Tables

**(a):** 

E-glass (0°)				
Layer orientation (°)	0°	45°	90°	−45°
Layer weight (g/m^2^)	472	—	45	—
Weight, knitting yarn (g/m^2^)	10			

**(b):** 

E-glass (−45°, 0°, 45°)				
Layer orientation (°)	0°	45°	90°	−45°
Layer weight (g/m^2^)	295	148	45	148
Weight, knitting yarn (g/m^2^)	12			

**Table 2:** Forces versus displacements of the fiberglass composite strips made by the E-glass (0°) and E-glass (−45°, 0°, 45°) with three different cutting directions (0°, 45°, 90°).

	Disp. (mm)	Test #1 (N)	Test #2 (N)	Test #3 (N)	Test #4 (N)	Test #5 (N)	Test #6 (N)	Test #7 (N)	Average (N)	Std (N)
E-glass (−45°, 0°, 45°)-0°	5	6.39	6.47	5.67	6.42	6.37	6.43	6.20	**6.28 **	**0.28 **
	7	9.04	8.92	7.90	8.86	8.80	9.10	8.65	**8.75 **	**0.41 **
	10	12.82	12.88	11.32	12.78	12.68	12.91	12.39	**12.54 **	**0.56 **

E-glass (−45°, 0°, 45°)-45°	5	4.23	4.61	3.67	3.84	3.92	4.10	4.07	**4.06 **	**0.30 **
	7	6.01	6.39	5.19	5.42	5.63	5.73	5.78	**5.74 **	**0.39 **
	10	8.51	9.20	7.37	7.70	7.89	8.20	8.18	**8.15 **	**0.59 **

E-glass (−45°, 0°, 45°)-90°	5	3.07	3.20	2.38	2.69	2.57	2.71	2.74	**2.76 **	**0.28 **
	7	4.40	4.38	3.59	3.78	3.65	3.75	3.91	**3.92 **	**0.34 **
	10	6.18	6.36	4.88	5.38	5.16	5.40	5.51	**5.55 **	**0.54 **

E-glass (0°)-0°	5	4.43	4.64	3.51	4.09	5.44	5.76	5.05	**4.70 **	**0.78 **
	7	6.25	6.47	4.89	5.78	7.80	8.12	7.13	**6.64 **	**1.14 **
	10	8.88	9.27	7.02	8.21	10.96	11.55	10.13	**9.43 **	**1.58 **

E-glass (0°)-45°	5	1.56	1.59	1.12	4.09	1.37	1.37	1.32	**1.77 **	**1.03 **
	7	2.23	2.26	1.60	5.78	1.95	1.99	1.91	**2.53 **	**1.45 **
	10	3.14	3.19	2.26	8.21	2.75	2.78	2.66	**3.57 **	**2.07 **

E-glass (0°)-90°	5	1.25	1.21	1.02	1.29	1.08	1.08	1.21	**1.16 **	**0.10 **
	7	1.74	1.72	1.47	1.85	1.67	1.50	1.67	**1.66 **	**0.13 **
	10	2.50	2.44	2.07	2.60	2.23	2.15	2.41	**2.34 **	**0.20 **

**(a):** 

Type	S-0	S-45	S-90	D-0	D-45	D-90
Estimated *E* (GPa)	19.90	13.03	9.17	11.01	13.03	18.06

Test #1	17.01	14.04	8.90	10.26	12.93	16.50
Error (%)	14.53	7.74	2.94	6.83	0.78	8.63
Test #2	18.67	12.72	8.99	10.99	12.65	18.37
Error (%)	6.19	2.39	1.92	0.15	2.93	1.72
Test #3	19.87	13.16	9.35	11.40	13.97	19.40
Error (%)	0.17	0.99	1.94	3.59	7.15	7.41
Mean	**18.52**	**13.31**	**9.08**	**10.88**	**13.18**	**18.09**
STD	**(1.44)**	**0.67**	**0.24**	**0.58**	**0.70**	**1.47**

Average error (%)	**6.95**	**2.12**	**0.98**	**1.15**	**1.18**	**0.17**

**(b):** 

Type	S′-0	S′-45	S′-90	D′-0	D′-45	D′-90
Estimated *E* (GPa)	25.5	8.78	7.18	23.88	8.78	8.8

Test #1	25.8	8.5	7.32	21.63	8.05	8.66
Error (%)	1.16	3.28	1.90	9.43	8.40	1.56
Test #2	27.16	8.70	6.81	25.53	8.39	9.06
Error (%)	6.5	0.92	5.15	6.91	4.52	3.02
Test #3	25.62	8.87	6.98	25.32	8.92	8.94
Error (%)	0.49	0.93	2.81	6.04	1.58	1.62
Mean	**26.19**	**8.69**	**7.04**	**24.16**	**8.45**	**8.89**
STD	**0.84**	**0.19**	**0.26**	**2.19**	**0.44**	**0.21**

Average error (%)	**2.72**	**1.03**	**2.00**	**1.17**	**3.72**	**0.98**

**Table 4:** The estimated and measured elastic modulus of the 6-layer composites.

Type	D222-0	D222-45	D222-90
Estimated *E* (GPa)	9.45	13.42	18.80

Test #1	10.34	12.85	19.06
Error (%)	9.38	4.26	1.37
Test #2	9.55	13.68	20.19
Error (%)	1.03	1.92	7.37
Test #3	10.20	13.92	18.76
Error (%)	7.92	3.77	0.21
Mean	**10.03**	**13.48**	**19.34**
STD	**0.42**	**0.56**	**0.75**

Average error (%)	**6.14**	**0.47**	**2.85**

**Table 5:** The estimated and measured elastic modulus of the semicircular composites.

	Estimated *E*	Measured *E*	Error (%)
E-glass (−45/0/45)-0	19.21	19.18	0.15
E-glass (−45/0/45)-90	9.42	9.22	2.05
E-glass (0)-0	26.75	26.84	0.37
S-0	19.90	20.42	2.60
S-90	9.17	9.98	8.79
D-0	11.01	10.89	1.08
D-90	18.06	20.71	14.64

**Table 6:** Specifications of the half-circular composite leg made by the E-glass (0°, 90°)-0°.

	Thickness (mm)	Width (mm)	Stiffness (k/m)	*E* (Gpa)
#1	3.60	20.69	2563.10	19.98
#2	3.60	20.86	2618.98	20.16
#3	3.61	20.41	2509.90	19.68
#4	3.60	20.90	2670.84	20.58
#5	3.62	20.77	2594.05	19.80

Averaged stiffness (k/m)			2591.37 ± 60.25	

**Table 7:** Specifications of the half-circular composite leg made by the E-glass (−45°, 0°, 45°)-90° and the E-glass (0°)-0°.

	Thickness (mm)	Width (mm)	Stiffness (k/m)	*E* (Gpa)	Estimated *E* (GPa)
#1	4.37	20.65	2708.81	11.83	10.19
#2	4.34	20.57	2664.14	11.64	
#3	4.15	19.95	2245.24	10.48	
#4	4.28	20.31	2455.86	11.23	
#5	4.35	20.83	2702.62	11.05	

Averaged stiffness (k/m)			2555.33 ± 201.95		

## References

[1] Alexander R. M. (1988). *Elastic Mechanisms in Animal Movement*.

[2] Blickhan R. (1989). The spring-mass model for running and hopping. *Journal of Biomechanics*.

[3] Holmes P., Full R. J., Koditschek D., Guckenheimer J. (2006). The dynamics of legged locomotion: models, analyses, and challenges. *SIAM Review*.

[4] Full R. J., Koditschek D. E. (1999). Templates and anchors: neuromechanical hypotheses of legged locomotion on land. *Journal of Experimental Biology*.

[5] Rahman M. H., Kittel-Ouimet T., Saad M., Kenné J.-P., Archambault P. S. (2012). Development and control of a robotic exoskeleton for shoulder, elbow and forearm movement assistance. *Applied Bionics and Biomechanics*.

[6] Controzzi M., D'Alonzo M., Peccia C., Oddo C. M., Carrozza M. C., Cipriani C. (2014). Bioinspired fingertip for anthropomorphic robotic hands. *Applied Bionics and Biomechanics*.

[7] Syrseloudis C. E., Emiris I. Z., Lilas T., Maglara A. (2011). Design of a simple and modular 2-DOF ankle physiotherapy device relying on a hybrid serial-parallel robotic architecture. *Applied Bionics and Biomechanics*.

[8] Folgheraiter M., de Gea J., Bongardt B., Albiez J., Kirchner F. (2009). Bio-inspired control of an arm exoskeleton joint with active-compliant actuation system. *Applied Bionics and Biomechanics*.

[9] Zhou X. D., Bi S. S. (2012). A survey of bio-inspired compliant legged robot designs. *Bioinspiration and Biomimetics*.

[10] Poulakakis I., Smith J. A., Buehler M. (2005). Modeling and experiments of untethered quadrupedal running with a bounding gait: the Scout II robot. *International Journal of Robotics Research*.

[11] Poulakakis I., Papadopoulos E., Buehler M. (2006). On the stability of the passive dynamics of quadrupedal running with a bounding gait. *International Journal of Robotics Research*.

[12] Smith J. A., Poulakakis I., Trentini M., Sharf I. (2010). Bounding with active wheels and liftoff angle velocity adjustment. *The International Journal of Robotics Research*.

[13] Saranli U., Buehler M., Koditschek D. E. (2001). RHex: a simple and highly mobile hexapod robot. *International Journal of Robotics Research*.

[14] Lin P. C. (2005). *Proprioceptive sensing for a legged robot [Ph.D. dissertation]*.

[15] Saranli U., Rizzi A. A., Koditschek D. E. (2004). Model-based dynamic self-righting maneuvers for a hexapedal robot. *International Journal of Robotics Research*.

[16] Johnson A. M., Koditschek D. E. Toward a vocabulary of legged leaping.

[17] Cham J. G., Bailey S. A., Clark J. E., Full R. J., Cutkosky M. R. (2002). Fast and robust: hexapedal robots via shape deposition manufacturing. *International Journal of Robotics Research*.

[18] Kim S., Clark J. E., Cutkosky M. R. (2006). iSprawl: design and tuning for high-speed autonomous open-loop running. *International Journal of Robotics Research*.

[19] Huang K. J., Huang C. K., Lin P. C. (2014). A simple running model with rolling contact and its role as a template for dynamic locomotion on a hexapod robot. *Bioinspiration & Biomimetics*.

[20] Moore E. Z., Campbell D., Grimminger F., Buehler M. Reliable stair climbing in the simple hexapod ‘RHex’.

[21] Campbell D., Buehler M. Stair descent in the simple hexapod RHex.

[22] Chou Y.-C., Yu W.-S., Huang K.-J., Lin P.-C. (2012). Bio-inspired step-climbing in a hexapod robot. *Bioinspiration and Biomimetics*.

[23] Campbell D., Buehler M., Siciliano B., Dario P. (2003). Preliminary bounding experiments in a dynamic hexapod. *Experimental Robotics VIII*.

[24] Chou Y. C., Huang K. J., Yu W. S., Lin P. C. (2015). Model-based development of leaping in a hexapod robot. *IEEE Transactions on Robotics*.

[25] Huang K. J., Chen S. C., Komsuoglu H., Lopes G. A. D., Clark J. E., Lin P. C. (2015). Design and performance evaluation of a bio-inspired and single-motor-driven hexapod robot with dynamic gaits. *ASME Journal of Mechanisms and Robotics*.

[26] Heglund N. C., Taylor C. R., McMahon T. A. (1974). Scaling stride frequency and gait to animal size: mice to horses. *Science*.

